# Beyond the first week: sustaining the feeling of social inclusion and sense of belonging for students

**DOI:** 10.1080/17482631.2024.2421032

**Published:** 2024-10-27

**Authors:** Arnfrid Farbu Pinto, Nina Petersen Reed, Odd Morten Mjøen

**Affiliations:** Department of Mental Health, Faculty of Medicine and Health Science, Norwegian University of Science and Technology (NTNU), Trondheim, Norway

**Keywords:** Student well-being, student mental health, interactions, emotional energy, interactional ritual change, introduction week

## Abstract

**Purpose:**

Promoting inclusive student communities and a sense of belonging is essential for university students’ well-being and mental health. Our aim in this study was to explore how universities can enhance student well-being by facilitating interactions that contribute to a sense of belonging within the university environment.

**Methods:**

Through thematic analysis of 309 written narratives collected from students at the Norwegian University of Science and Technology (NTNU), we explored significant events related to their studies, social lives, or personal experiences.

**Results:**

Introduction Week is important for students’ well-being, providing a key opportunity to form initial social connections and foster a sense of belonging. However, its impact is inconsistent, and building lasting relationships requires continued effort throughout the academic journey. Recognizing and addressing potential challenges when expectations are unmet is essential for enhancing student belonging.

**Conclusions:**

Our findings suggest that while Introduction Week plays an essential role in breaking social barriers and ensuring immediate social inclusion, the greatest challenge lies in establishing structures that facilitate belonging and relationships throughout the entire period of studies. Recognizing the diverse needs of students, universities should offer a variety of platforms for engagement and connection to enhance student well-being.

## Introduction

Becoming a student can represent an exciting and important phase in life. It’s a time for self-exploration and learning, but also a time to take greater responsibility for one’s own life and well-being. While universities offer numerous opportunities for personal and academic development, being a student can also present some challenges. A series of studies have examined this issue, revealing that a substantial number of students are struggling with mental health issues (Auerbach et al., 2016; Ibrahim et al., 2013; Larcombe et al., 2016; Sheldon et al., 2021). In line with this, there’s been a noticeable increase in the demand for university counselling services (Broglia et al., 2018; Xiao et al., 2017). The growing awareness of students’ mental health issues has increased the interest in the specific challenges they encounter.

University efforts to assist students struggling with mental health have previously focused on a reductionist and disease-oriented approach. However, recent trends indicate a shift towards a more holistic approach. This shift includes creating supportive environments that promote mental health and well-being through prevention, fostering a sense of belonging, and community engagement (Dooris et al., 2022). Student well-being has gained increased attention as a priority area within educational policy and practice, and efforts to measure and monitor student well-being have intensified (Hossain et al., 2023). Although there is currently no consensus on which specific aspects of student well-being are the most relevant for exploration and understanding (Hossain et al., 2023), recent research has increasingly pointed to a sense of belonging as a significant factor. Studies have shown that a sense of belonging in higher education is crucial for students’ mental health and well-being, being associated with reduced loneliness, lower levels of depression and anxiety, and improved academic performance (Allen et al., 2021; Campbell et al., 2022; Dietz et al., 2023; Haslam et al., 2016; Meehan & Howells, 2019; Nunn, 2021; van Herpen et al., 2020). In this context belonging is considered a fundamental human need. According to Dietz et al. (2023) Maslow (1954) identifies belonging as an essential component of his hierarchy of needs, where individuals seek to be a part of a group. Additionally Baumeister and Leary (2017) highlight the importance enduring, positive relationships for human well-being. Research indicates that when students feel a sense of belonging, their engagement increases, academic performance improves, and they experience greater personal well-being (Dietz et al., 2023). This sense of belonging also encourages active participation, which in turn leads to better outcomes and further enhances personal well-being, creating a positive feedback loop for both academic and personal lives (Dietz et al., 2023). Additionally, a sense of belonging not only enhances students’ integration into university life but also acts as a powerful protective mechanism against stress and mental health challenges (Walton & Cohen, 2007).

Following this, creating an environment where students feel they belong is important. According to Allen et al. (2021) this requires fostering successful interactions on multiple levels: among students, between teachers and students, and within the broader university community. Meehan and Howells (2019) highlight that social interactions are essential for students transitioning to higher education, as support from peers, friends, housemates, and academic staff helps them feel connected and capable, thereby fostering a sense of belonging. This is supported by Maunder (2018), who points out that strong social interactions with peers are crucial for successful adjustment to university life, and by Pittman and Richmond (2008), who find that the quality of these interactions, particularly friendships, and a sense of belonging to the university, are positively correlated with students’ psychological adjustment during the transition to college. As such, facilitating social interactions to promote a sense of belonging appears important for students’ successful transition and adjustment to university life.

Introduction Week is promoted as an effective early measure in a student’s university transition, aimed at enhancing social integration and establishing a solid foundation for their sense of belonging (Murray, 2022; Myrtveit et al., 2017; Thomas & Hanson, 2014). The importance of such early intervention is supported by several previous studies, including Engle and Tinto (2008), which point out that for first-generation students, it is especially important as they face multiple social and academic challenges. Student mentors play a pivotal role in this integration process by facilitating social interactions, helping new students form friendships, and sharing knowledge, thereby creating a supportive environment for academic and social adjustment (Strayhorn, 2018). These efforts are further complemented by successful interactions with peers and involvement in student activities (Strayhorn, 2018). Active participation in student activities is essential for enhancing students’ well-being, fostering meaningful interactions, and strengthening the sense of belonging (Bates et al., 2019; Campbell et al., 2022; Dietz et al., 2023; Pittman & Richmond, 2008). Walton and Cohen (2007) find that marginalized students are more sensitive to exclusion in such activities, leading to doubts about belonging and reduced motivation. This heightened sensitivity underscores the importance of Strayhorn (2018) assertion that student leaders play an essential role as facilitators and door openers. Therefore, fostering a supportive university environment through early interventions like Introduction Week, active participation in student activities, and the involvement of student mentors can enhance students’ social integration, sense of belonging, and overall well-being.

Despite the recognized benefits of early intervention programmes like Introduction Week and the involvement of student mentors, there remains a significant gap in understanding the specific elements that contribute to their effectiveness and the long-term impact on student well-being. Thomas and Hanson (2014) highlight the need for more detailed research on social integration activities and their direct impact on student well-being. Myrtveit et al. (2017) emphasize the need for further research on long-term effects and reasons for non-participation. Although prior studies have addressed elements such as group identity, supportive learning environments, and positive relationships with academic staff and peers, there is limited understanding of how specific social interactions contribute to creating and maintaining a sense of belonging (Allen et al., 2021; Baik et al., 2019; van Herpen et al., 2020). This highlights the need for further research into the fundamental processes that contribute to feelings of belonging, as well as the strategies that can enhance these feelings. By understanding these dynamics and contextual conditions better, we can enhance the university environment to improve students’ sense of belonging and overall well-being. To explore these dynamics further, we turn to the theoretical framework that supports our study.

## Theoretical framework

In this study, we explore social interactions within university environments, making use of Collins (2004) interaction theory in our analysis and interpretations. This theory provides a framework for understanding how social interactions influence an individual’s feeling of well-being. It specifically emphasizes how such interactions can generate emotional energy, which in turn affects a person’s sense of belonging, motivation, engagement and well-being.

Collins builds on the classical interaction theories previously described by Durkheim (1915) and Goffman (1955). Durkheim, as discussed in Collins (2004) underlines the importance of social structures, norms and rituals for individual behaviour and societal coherence. Durkheim points out that belonging to a group is central to a person’s identity and their perception of their role in society. He believes that social structures and collective beliefs, such as norms, values, and trust, are key to shaping an individual’s behaviour and consciousness. When a group of people come together around a shared activity, they experience a physical and emotional synchronization. This creates a strong sense of unity and awareness, which Durkheim refers to as “collective effervescence”.

Goffman’s work, also as interpreted by Collins (2004), focuses on the micro-level of society and how people fulfil social expectations and norms through rituals. He views social order as a constant movement, pointing out that the roles people play in society are not fixed roles but roles that evolve in different situations. Collins (2004) extends the ideas of Durkheim and Goffman, examining how individuals achieve emotional energy through interaction with others. Successful interactions result in high positive emotional energy, strengthened bonds, and loyalty to the group, a phenomenon he calls interaction ritual chains. Collins underscores the significance of everyday interactions and how the transmission of symbols, cultural values, and norms during these encounters generates emotional energy. When this transmission aligns with the recipient’s own values or emotions, it deepens their sense of belonging and reinforces their connection to the community. Key conditions for such rituals include physical presence of individuals, a shared focus, and a shared mood. With this theoretical framework established, we now turn our attention to the research question and how these dynamics unfold in practice.

## Research question

Inspired by Collins interaction theory (Collins, 2004), this study examines how social interactions influence students sense of belonging and well-being. Our aim was to explore how universities can enhance student well-being by facilitating interactions that contribute to a sense of belonging. We addressed this by analysing and interpreting students’ own narratives from their time as students, whether related to studies, social life, or personal experiences. The research question guiding our analysis was: What kinds of interactions and events do students describe as having a positive or negative impact on their well-being and sense of belonging within the university environment?

## Method

This study is based on narrative data collected by Study Trondheim in the project “In My Experience” (inmyexperience.no). The overall goal of “In My Experience” was to explore and understand what affects students’ quality of life, through a methodical approach that combined qualitative and quantitative research data. Following approval from SIKT—Norwegian Agency for Shared Services in Education and Research (104826), students at NTNU (Norwegian University of Science and Technology) were invited to share their firsthand experiences from student life. An online survey, which took approximately 10–15 minutes to complete, invited students to share personal stories with the following prompt:

“To get started, think of something that happened in your life as a student, something that comes to mind now and that has meant something to you. It might be related to your studies, your social life or your personal life—something positive or negative, something big or small. Make your description as short or long as you want”.

Students wrote down their narratives before moving on to a form with 27 different questions about their life situation. Our research specifically focuses on the qualitative narratives, alongside demographic information such as gender, age, and years of study. Additionally, students were asked to assess the impact of their experiences, rating them as very positive, positive, neutral, negative, or very negative; this evaluative data is also briefly addressed in our study. The narratives were collected from October 2020 to October 2023, with the most responses received between October 2021 and April 2022. A total of 604 narratives were initially collected. We decided to exclude data where students explicitly linked their experiences to COVID-19, as this period was marked by unique challenges like social distancing and the shift to online learning. These conditions, fundamentally altering the educational landscape, do not reflect typical academic settings, which usually involve physical interactions. By removing these narratives, we retained only data that represents more standard interactions within the learning environment, aligning with our focus on understanding the dynamics of a typical educational setting. This exclusion left us with 470 narratives. Among these, 351 reported positive or very positive experiences, while 95 described negative or very negative experiences. Among the respondents, 292 were women and 169 were men. The age distribution among the women and men who responded was relatively even, ranging from 18 years to 30 years or older. The students were also evenly distributed in terms of the number of years of study, from first-year students to students in their seventh year of study. 309 of the 470 narratives were about everyday interactions in the educational setting, and because of their potential contributions to our aim, these were the ones we focused on in our analysis and interpretation of the data.

The participants have consented to the terms of data collection and usage, which consists solely of anonymized information gathered through SenseMaker® software, controlled by Study Trondheim, and to which Study Trondheim has granted us access. Access to the survey data is detailed at (https://www.inmyexperience.no/no/personvern). With the absence of person-specific data, both the Data Protection Officer at the Norwegian University of Technology and the Norwegian Agency for Shared Services in Education and Research (Sikt) confirmed that further ethical clarification was not necessary.

We did a reflexive thematic analysis of the data, inspired by Clarke and Braun (2021). The analysis began with an inductive approach where all three authors immersed themselves in the data, repeatedly reading the narratives, followed by initial coding. Preliminary findings were then shared and discussed among the co-authors. Moving forward from step one, we further explored the coding, and ended up with 176 different codes for classifying the students’ narratives related to interactions in student life. The authors understood 114 of these codes as related to some form of community. Other important common terms were friendship, social belonging, mastery, and relationships.

In the next analytical step, we name and analyse thematic patterns. This provided a deeper understanding of how the data could be interpreted. The first author, in collaboration with the co-authors, carefully revised and refined each theme to accurately reflect both the data and the emerging theoretical insights. Based on the preliminary findings, the authors identified Collins’ theories on interaction ritual chains as an interesting theoretical framework for further analysis (Collins, 2004). The preliminary revised findings were discussed among the co-authors in an interactive dialogue between data, previous knowledge, and theory. We worked through various thematic divisions, breaking them down into sub-themes, and moved citations from the narratives between themes until we identified themes that accurately described our findings and provided a comprehensive picture of the data. The chosen themes were theoretically influenced, highlighting interaction ritual chains and their role, as suggested by Collins (2004).

From our analysis, we identified four key categories that collectively reflect the essence of the students’ narratives related to our aim: The first category, “Finding one’s group: Well-being in student life,” explores the fundamental role of supportive groups in enhancing student well-being. The second, “Starting the academic journey with social success: New acquaintances and an immediate sense of belonging,” focuses on the initial interactions that foster an immediate sense of belonging. The third, “From new acquaintances to an enduring sense of belonging,” examines the transition from initial connections to lasting social structures. Lastly, “The student journey: When students’ social expectations and needs are unmet,” addresses the challenges students face when their social expectations are not fulfilled. In the following sections, we will present our findings.

## Findings

Our findings support previous studies, demonstrating that establishing secure relationships and a sense of belonging is essential for students’ well-being. Our findings also highlight the importance of interactions, particularly those that occur during Introduction Week and through various activities and events, in forming these relationships and fostering belonging. When these interactions are successful, students’ well-being improves, but when they fail, it can have lasting negative consequences. Furthermore, we demonstrate how these successful interactions can be achieved through various arenas and social activities, such as student politics, theatre groups, shared housing, student associations, sports, and volunteer work. These arenas and activities become available to students in different ways and to different extents, presenting them with both positive and negative challenges and experiences, as we will show in the following.

## Finding one’s group: well-being in student life

Our findings underline the importance of establishing a robust foundation for building relationships and fostering successful interactions in student life. This significance is vividly illustrated in a narrative shared by a student, who felt privileged to have formed strong relationships early in her academic journey: “I was fortunate to get to know many people at the beginning of my studies. Being part of a group of friends and a community improved my self-confidence and overall well-being. I no longer feel lonely” (Female, age 18–21). This statement clearly emphasizes that establishing relationships and engaging in successful interactions can enhance well-being. Similar experiences of establishing friendships and well-being are also described by other students who have participated in different student activities, where they interacted with people with similar interests. For instance, this student participated in the student choir:

When I was new to Trondheim, I got involved early on at “Samfundet” through the choir. This has been very important for my well-being in Trondheim up to now, as it’s where I’ve formed most of my friendships, and where I spend most of my time outside of school. (Female, age 18–21)

A common theme in these stories is the importance of belonging to a group of people, having a unifying place, and engaging in shared activities. Whether it’s at the student society, film club, choir, collective, or in politics, it’s through these collective experiences that relationships, joy, security, and friendships emerge. The students describe a sense of community that they find invaluable, as expressed by this student: “I didn’t realize how wonderful it was until I became a part of it, but now I thank myself every day for doing so. You get to feel a sense of community and belonging that is truly priceless” (Female, age 22–25).

A student describes how small, random interactions can trigger significant changes in life and influence future events. He reflects on how volunteer work at the student culture house, “Samfundet”, has played a major role in his life have led to both friendships and enriching experiences:

Looking back at my time as a student, I can’t imagine what it would have been like without “Samfundet”. Most of my friends, my biggest highs, and lowest lows, have I experienced throught volunteer work at “Samfundet”. This gets me thinking about the small, random interaction in life that can put you on a trajectory with enormous impact on your life. This is truly frightening and amazing at the same time. (Male, age 22–25)

This student shares a story about what he describes as small random interactions with others can trigger significant changes in life and influence future events. Through active participation in student volunteering, he has not only formed close friendships but also gathered numerous memorable experiences that have enriched his student life.

Thus, the students’ narratives highlight the importance of early connections, seemingly random interactions, active participation, and the diverse opportunities available throughout their academic journey. Whether through volunteer work, student societies, or shared activities, these experiences foster a sense of belonging that is fundamental for their well-being. The students describe that their sense of belonging to the university increases when they find a group of peers they enjoy being with. In the following categories, we will take a closer look at the path leading to such meaningful interactions and how they can impact students’ lives in both the short and long term.

## Starting the academic journey with social success: new acquaintances and an immediate sense of belonging

The initial weeks of university, especially Introduction Week, play a key role in forming students’ immediate sense of belonging. This is vividly illustrated through the students’ detailed descriptions of events and activities that took place during the first weeks. These experiences seem to lay the foundation for their continued engagement and well-being at the university. In many of the narratives we analysed, students describe the beginning of their academic journey as a positive experience, where they experienced early turning points in their emotional state and perspective. One student describes how initial nervousness about moving to a new place and meeting strangers quickly transformed into excitement and anticipation for an amazing study period. It’s a moment that seems to stand out as a turning point, filling the student with optimism for the future:

One of my favorite memories from my short time as a student is from the introduction week. How the nerves of moving to a new place and meeting many unknown people simply vanish during the few days of the introduction week, replaced by an expectation that the study period would be absolutely fantastic. (Male, age 18–21)

The students’ narratives also reveal that the introduction week may be a significant highlight, being a moment in their academic journey where they experience an immediate, and perhaps surprising, sense of belonging to a student community as illustrated by this student’s story: “When the entire hall began to sing, as loudly and off-key as they pleased, I suddenly realized that I was part of something bigger, and what I had been searching for all along” (unspecified, age 22–25). We understand that this moment, where everyone participates in a spontaneous song, triggered intense joy for the student and creates an immediate sense of belonging. Further, a sense of gratitude seems to be a common response for many students’ to their experiences during the introduction week, as exemplified by one student:

I want to give a big thank you to everyone who contributes to making the introduction week what it is today. Personally, I believe this is one of the best arrangements to provide new students with the best introduction to a new city, a new study program, and, most importantly, new friends. (Male, age 18–21)

Our findings highlight the pivotal role of Introduction Week in fostering an immediate sense of belonging among students. The narratives reveal that this period is not only a time of initial adjustment but also a transformative phase that sets the tone for students’ academic journey. The experiences shared by students highlight how successful interactions and significant events during these early weeks can alleviate initial nervousness, replacing it with excitement and optimism.

## From new acquaintances to an enduring sense of belonging: the students’ social journey

While the Introduction Week plays a central role in the beginning of the students’ journey, it’s not guaranteed a lasting sense of belonging. Sometimes the acquaintances from the introductory week remain just that—acquaintances. Our findings show that developing further these initial relationships requires active effort and can occur through various avenues throughout the academic journey. Some students take initiatives themselves to strengthen these bonds, while others may need additional support. One student illustrates this by emphasizing the need for active participation: “You have to be proactive and engaged to get to know people. One of my classmates took the initiative to introduce themselves, and after we started talking, we’ve been sitting together in every lecture, developing a meaningful friendship” (Female, age 18–21).

Further, our findings show how a sense of belonging may be further developed beyond the Introductory Week by students taking the opportunity to engage in various activities with others. Some students discover these opportunities of social interactions through flyers or invitations posted by student associations during Introduction Week, while others highlight the role of student mentors during this transition period. For instance, one student shares her experience:

On the first day, I was pleasantly surprised when the leader of the student association created a group chat for all new students and invited us to a pre-party in the evening … Now we have a fantastic class environment, and we do things together almost every week. (Female, age 22–25)

Student mentors also encourage participation in various student activities such as student associations and sports. For example, one student was influenced by his mentor to join the soccer team for their study programme, which led to lasting friendships:

I was convinced by my mentor to join the soccer team for our program! On the team, I found several of the friends I have today. I probably wouldn’t have gone to that practice if my mentor hadn’t said she was going too, so we could go together. (Male, age 22–25)

The role of student mentors in fostering an enduring sense of belonging is highlighted by several, as they provide valuable information and actively encourage participation in student activities. Our findings further show that university-organized events may also have an important role in fostering social interaction by creating opportunities for informal gatherings. Even small everyday joys, like free oatmeal in the morning or “cinnamon bun Wednesdays”, contribute to creating social bonds, as well as positive emotional experiences. These events can also motivate students to attend school instead of staying home, as this example shows:

My schedule last fall included a full day of classes on Wednesdays, which was quite demotivating and tough. But then we discovered that the campus café had cinnamon bun Wednesdays. It became a weekly highlight, and it was both social and motivating. I chose not to skip on certain Wednesdays because I knew it was “cinnamon roll Wednesday”. (Female, age 22–25)

In the learning environment, excitement about meeting friends in class is expressed: “When I started my studies, I had no idea how much the people I met along the way would mean to me. Some of them are now my closest friends, and I look forward to seeing them in lectures” (Female, age 22–25). The role of the lecturer in this context is also highlighted:

I had a lecturer during my first two years of studies who went the extra mile to create a positive class environment and build good relationships with us students. Even something as simple as her saying hello to everyone and welcoming us to the lecture made me feel seen and included by her. (Male, age 22–25)

Our findings indicate that while the Introduction Week plays an important role, the development of lasting relationships requires continuous effort and engagement. Mentors and university staff play significant roles in encouraging participation in student activities and by facilitating student encounters. Events and activities organized by student organizations, along with initiatives through the cafeteria or by engaged and interested lecturers, contribute to creating social bonds and positive experiences, which students describe as having an impact on their well-being and sense of belonging within the university environment.

## The student journey: when students’ social expectations and needs are unmet

In the preceding categories, we have explored how students form friendships and a sense of belonging, either immediately through Introduction Week or with the help of mentors or university staff. This support helps integrate them into stable groups, contributing to their overall well-being. However, not all students have the same experience. Some students struggle to achieve this sense of belonging, often starting early in their university journey. We will now examine the narratives where students initially felt a sense of exclusion, which for some, led to long-lasting social consequences. One student describes how illness prevented participation, resulting in exclusion and a first year of studies without social contact:

I didn’t attend the introduction week more than twice due to illness. The introduction week started on Monday, but I showed up on Thursday night when everyone was very drunk. I got scared because everyone knew each other except me, so I went home. The next time I attended was either Tuesday or Wednesday in the second week of the introduction. By then, the others were almost sober. I got to know some new faces, but it was very difficult to break into the group dynamic. After two hours, I forced myself to stay there and not run away. Unfortunately, this means I’ve gone through my first year of studies without talking to anyone. (Female, age 22–25)

Similarly, a senior student described how he felt excluded by her fellow students. Consequently, feelings of isolation arose, as he was deprived of the social student life he wished for: “I am a relatively mature student and have always wanted to participate in social activities. However, it hasn’t been easy to achieve, and as a result, I’ve felt somewhat left out … ”(Female, age 38 or older).

Further, some students experienced difficulties when they did not follow the norms of the introduction group, such as expected alcohol consumption. One student, an elite athlete, chose to avoid alcohol, which led to a lack of inclusion in social events:

I learned that one should not skip drinking during introduction week. I chose not to drink at the home parties we had. Those home parties were the only things we did during introduction period apart from a meeting outdoors, without any formal program. I only drank soft drinks, except for (legit) a couple of beers on a few evenings (never got drunk). (I am an elite athlete at the national level in sports). I quickly realized that there were other parties within my introduction group that I hadn’t heard about. (Male, age 18–21)

Several other narratives communicate similar experiences, where a lack of success during the introduction week has had a significant impact on their current experience of student life. For instance, one student felt friendless for the first time during the introduction week, which most students consider a vital time for making friends: “The first thing I think of is introduction week, and in that exact moment, I realized that for the first time in my life, I had become the person without friends” (Female, age 22–25). The narratives indicate that social roles are often formed during introduction week. Exclusion from these early-formed communities can lead to feelings of loneliness, as illustrated by this student: “ … there’s nothing worse than showing up to a lecture and being the only one in the entire room who has to sit alone” (Female, age 22–25).

Our findings highlight that while some students successfully integrate socially and academically, others face challenges that can lead to feelings of exclusion and loneliness. When students’ social expectations and needs are not met early on, this can result in long-term social consequences. The social roles established during Introduction Week can set the tone for the rest of their time at university, and students who fail to find their place in these initial groups may experience a persistent sense of isolation and lack of belonging.

## Discussion

This study explores how universities can enhance student well-being by facilitating interactions that contribute to a sense of belonging within the university environment. To ground this discussion, we use Collins (2004) interaction ritual chains theory as a framework. According to Collins, social interactions follow a ritualistic pattern where emotional energy is generated through shared experiences, common focus, and group solidarity. These interactions are key to strengthening bonds and fostering a sense of belonging. Successful interactions, which align with individuals’ values and emotions, generate high emotional energy, motivating further engagement and reinforcing the social ties within the group. This theory provides a valuable lens for interpreting how students’ interactions contribute to their sense of belonging and overall well-being. By examining the ways in which these interactions contribute to students’ sense of belonging, we seek to uncover strategies to achieve and improve these vital connections. Our analysis and interpretation of students’ narratives resulted in four key findings: The importance of supportive groups for well-being, the role of early social interactions in fostering a sense of belonging, the transition from initial connections to lasting social structures, and the potential pitfalls when expectations are unmet. Collins (2004) theory helps explain how emotional energy from successful interactions enhances well-being and supports students’ integration into university life.

Our findings emphasize that connecting with a group where consistent, successful interactions take place is key for generating the emotional energy that enhances well-being. This is evidenced by the significant increase in well-being observed when students establish peer connections, as one student noted: “One of the best things that happened to me during my studies was meeting like-minded people and finding a sense of belonging in social groups”. This highlights how early group formation boosts student engagement and well-being. Furthermore, this aligns with previous research, which emphasizes that establishing connections with peers is vital for fostering a sense of belonging and integration within the university environment (Allen et al., 2021; Baik et al., 2019; Dietz et al., 2023; Maunder, 2018; Pittman & Richmond, 2008; Strayhorn, 2018) Finding one’s group is often described as a challenging yet important aspect of the university experience (Maunder, 2018), a conclusion also supported by our study. We found that while some students establish these connections within the first weeks, others require additional time and support from their peers and the university staff.

Introduction Week emerges as a pivotal time for students to establish social connections. It provides a unique opportunity for students to meet peers in an informal setting, laying the foundation for them to find their group and build lasting relationships. Our findings align with previous research (Myrtveit et al., 2017; Strayhorn, 2018; Thomas & Hanson, 2014), showing that this period significantly influences students’ sense of belonging. Serving as a “kickoff” event, Introduction Week allows students to connect with peers and mentors in informal settings that promote camaraderie and joy, thereby shaping their overall perception of the university experience. From Collins’ perspective, Introduction Week functions as a powerful interaction ritual where shared activities generate emotional energy that strengthens bonds among participants. We also draw on Durkheim’s concept of effervescence, to describe the powerful impact that social events can exert by capturing the collective energy and enthusiasm that arises within social groups during events like Introduction Week. Through these shared experiences, participants develop a stronger connection to each other and to the group as a whole (Collins, 2004). The initial sense of belonging begins here, as students immediately bond with fellow students.

Our findings also highlight the critical role of student mentors in this process. By sharing essential knowledge about the university, events, opportunities, and cultural values, mentors facilitate student integration, a conclusion supported by earlier studies (Engle & Tinto, 2008; Strayhorn, 2018). The role of student mentors in relation to new students, which Collins (2004) characterizes as the “transmission of symbols,” allows for the sharing of knowledge about organizations, events, and opportunities, thereby fostering common ground and a sense of belonging among newcomers. Additionally, Goffman’s concept of performance (Goffman, 1955) offers further insight, explaining how, through observation and imitation, experienced students demonstrate how to navigate the “stage” of university life, enhancing the sense of belonging for newcomers and contributing to the formation of strong, inclusive communities.

Many of the student narratives we analysed describe the joy of being part of an established group, whether they start in the student choir, take on a role in student politics, or join the student handball team. Finding one’s group provide a sense of belonging and friendship, continuing the “wow effect” from the introduction week. Campbell et al. (2022) emphasize the importance of shared communities, describing them as key for student well-being. They highlight how positive emotional cycles arise through social interactions and shared achievements, promoting mental health and strengthening social bonds among students. This aligns with Collins’ theory of interaction ritual chains, which suggests that repeated successful social interactions not only enhance individual feelings of success but also foster a sense of belonging. Strayhorn (2018) supports this view, arguing that such interactions are essential for developing a strong sense of belonging. Thus, the establishment of strong chains of interaction within a supportive community can have a significant positive impact on students’ overall well-being.

Thomas and Hanson (2014) explores the transition from Introduction Week to the establishment of secure networks, highlighting the importance of these initial weeks for students in fostering lasting connections that enhance their sense of belonging and well-being. Murray (2022) and Strayhorn (2018) both emphasize that challenges faced during this initial period can lead to feelings of isolation and discomfort, potentially resulting in reduced motivation and marginalization. In our study, we find similar results, where students describe feelings such as exclusion and loneliness that arise early in their academic journey and seem to become persistent throughout their studies for some. Although Introduction Week aims to be inclusive, our findings reveal that factors such as physical absence or alcohol avoidance can lead to exclusion and feelings of unmet expectations.

Collins (2004), building on Goffman’s theories, provides insight into how feelings of exclusion can arise during events intended to be inclusive. He explains that group’s function based on implicit rules that promote solidarity and identity, but these rules can also lead to some students feeling excluded. Collins (2004) further elaborates on the negative emotional energy that arises when students’ expectations are unmet or when they feel excluded from group activities. When group activities and behaviours do not align with a student’s values or norms, it can lead to negative emotions and discourage future engagement. For example, students who participate less in social activities or abstain from alcohol may feel disconnected from the broader community, complicating the transition from Introduction Week to forming stable social networks. Therefore, it is important to create more opportunities for these students to engage with their peers. This necessity becomes even more evident when considering the potential pitfalls, as feelings of exclusion can deepen if students fail to find their place early on.

Building on these findings and prior research, this study emphasizes the importance of universities creating robust opportunities for social networking. It is essential to establish a sense of belonging and community for all students beyond the initial week—particularly for those who may not immediately feel connected and might need additional support to fully integrate into university life. We recommend that university management, in collaboration with student organizations, take a leading role in making Introduction Week both inclusive and engaging for all students. This event is essential in helping students begin their journey of finding their group, establishing an immediate sense of belonging, and facilitating their transition into stable, supportive social circles. Faculties can further enhance inclusion by expanding mentors’ responsibilities and providing them with the necessary training to understand the mechanisms that may lead some students to feel excluded. With this knowledge, mentors can actively work to create more inclusive activities. Additionally, we suggest implementing extended mentor support, where experienced students guide newcomers over an extended period. Given the significant role that these mentors play, extending the mentoring period through the first year can be particularly beneficial for students who struggle to make connections. Moreover, all university staff should actively contribute to creating an inclusive atmosphere by warmly welcoming students, showing genuine interest, and facilitating opportunities for them to interact with one another. By offering ongoing opportunities for engagement, universities help ensure that every student can form meaningful connections, which are vital to their academic success and overall well-being. This coordinated effort, which extends beyond Introduction Week, is crucial for maintaining the positive impact of early social interactions and ensuring that everyone finds their place within the university environment.

## Conclusions

Our study illustrates the impact of successful interactions on student well-being, offering actionable insights for higher education institutions. By fostering environments that creates emotional energy and mastery through interaction rituals chains, universities can significantly enhance the student experience. Key moments, such as introduction week, mentorship programmes, and inclusive activities, play a pivotal role in establishing a sense of community and belonging. Recognizing the diverse needs of students, universities should offer a variety of platforms for engagement and connection. Proactive steps to include all students can create an environment where individuals not only are present but also thrive, growing both personally and academically. Promoting successful interactions enables students to build strong bonds, increase their emotional energy, and enhance overall well-being.

Future research should examine how university environments and the relationships between university staff and students in enhancing student well-being. Our findings suggest that it’s essential for students to feel acknowledged and seen, not only by their peers but also by the university staff, emphasizing the importance of their visibility and significance in the student environment. By addressing these elements, we can take steps towards a more caring and inclusive academic environment.

## Limitations

The study has certain limitations, including not considering how individual differences and experiences influence perceptions and outcomes. Some students may struggle with group activities, preferring independent relationship-building. The study’s context-specific nature may limit its generalizability. Although the survey that the students responded to was open to everyone, it’s possible that a significant portion of recruitment occurred through social relationships, such as student volunteering. This group of students may be particularly interested in social activities and relationship build. Acknowledging and exploring these limitations in future research is crucial for a nuanced understanding of social interactions and student well-being.
