# Correction

**DOI:** 10.1080/17482631.2024.2425566

**Published:** 2024-11-06

**Article title**: Beyond the first week: sustaining the feeling of social inclusion and sense of belonging for students

**Authors**: Arnfrid Farbu Pinto, Nina Petersen Reed and Odd Morten Mjøen

**Journal**: *International Journal of Qualitative Studies on Health and Well-being*

**Bibliometrics**: Volume 19, Number 01, pages 1-11

**DOI**: https://doi.org/10.1080/17482631.2024.2421032

It has been noted by the authors that the order of author list should be changed to **‘**Arnfrid Farbu Pinto, Odd Morten Mjøen and Nina Petersen Reed’. This correction has not changed the description, interpretation, or the original conclusions of the article. The authors apologize for any inconvenience caused.

